# Liver hydatid cyst with cutaneous fistulization to the right breast: a case report, management, and literature review

**DOI:** 10.1002/ccr3.999

**Published:** 2017-05-22

**Authors:** Mansour El Khoury, Antoine El Asmar, Wissam Dib, Elie Creidi, Madeleine Yehia, Imad Hajj

**Affiliations:** ^1^ Department of Clinical SurgeryGeneral and Digestive SurgerySaint Georges Hospital University Medical CenterBeirutLebanon; ^2^ Faculty of MedicineUniversity of BalamandBeirutLebanon; ^3^ General and Digestive Surgery ResidentSaint Georges Hospital University Medical CenterBeirutLebanon; ^4^ Saint Georges Hospital University Medical CenterBeirutLebanon

**Keywords:** Cutaneous fistula, hydatid cyst complications, hydatid cyst management

## Abstract

*Echinococcus granulosus* is the most common tapeworm causing hydatid disease in humans. Its least‐encountered complication is cutaneous fistulization. Omentoplasty, cyst, and fistulous tract drainage revealed successful in the management of this complication. Such intervention can be an alternative when conservative management fails, and a minimally aggressive procedure is required.

## Introduction

*Echinococcus granulosus* is the most common tapeworm causing hydatid disease in the human species 1. The most commonly affected organ is the liver in 52–77% of cases 2. The most encountered complication is rupture and communication between the cyst and the biliary tree 3. The least‐encountered complication or presentation is a hydatid cyst – cutaneous fistula, with only 17 cases reported in the literature 4. We present a case of a liver hydatid cyst complicated by cutaneous fistula, successfully treated by omentoplasty, cyst drainage, and fistulous tract drainage.

## Case Report

This is a case of a 68‐year‐old female patient, known to have coronary artery disease and multiple metabolic disorders, who presented for the management of a right breast cutaneous fistula.

Four months prior to that our patient underwent a partial mastectomy for a fibroadenoma in her right breast that was complicated by a wound infection and persisted despite medical treatment, antibiotics, and superficial drainage. The wound had continuous purulent discharge.

**Figure 1:**
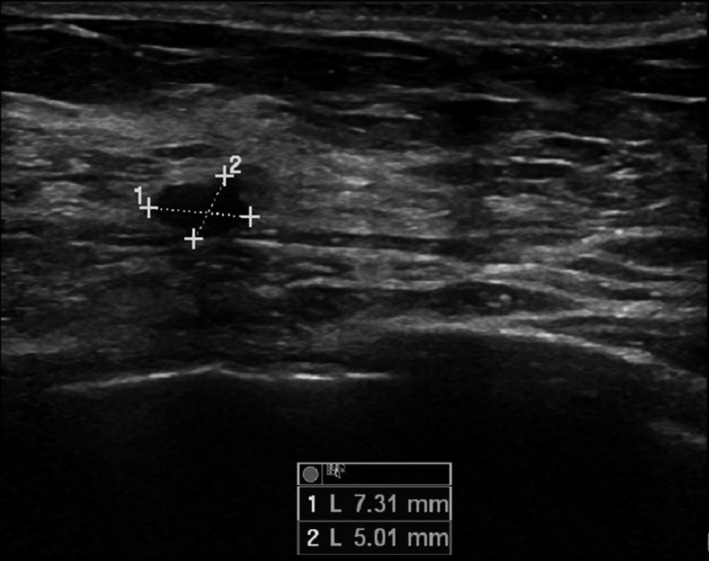
Ultrasound, coronal cut, showing the 5 × 7 mm lumen of the fistulous track.

**Figure 2:**
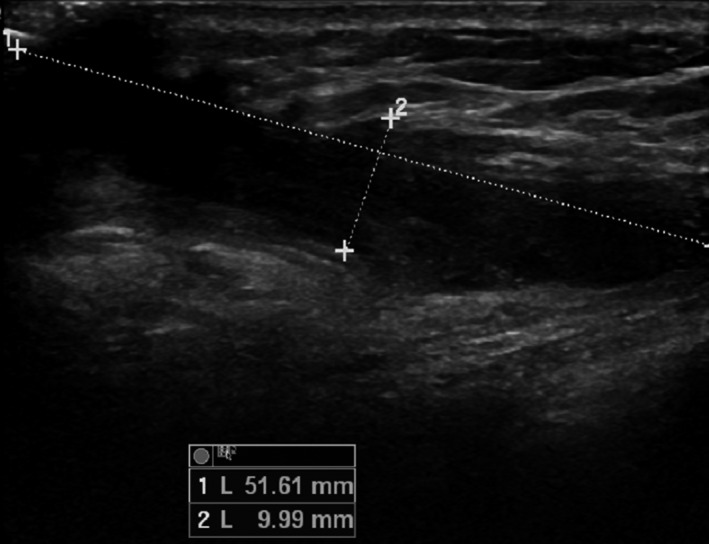
Ultrasound, sagittal cut, showing the 5 × 10 cm length of the fistulous track.

Ultrasound performed (Figs 1 and 2) showed a fistulous tract reaching the muscular layer of the chest in the left lower quadrant of her right breast.

Laboratory studies carried out were normal except for a mildly elevated CRP: hemoglobin 12.4 g/dL, hematocrit 37.0%, white blood cells 7000/mm^3^, neutrophils 68.2%, platelets 283,000/mm^3^, SGPT 32 U/L, SGOT 16 U/L, direct bilirubin 0.09 mg/dL, total bilirubin 0.23 mg/dL, alkaline phosphatase 106 U/L, *γ*GT 48 U/L, and *CRP 4.27 mg/dL*.

A culture taken from the breast discharge revealed growth of *Escherichia coli* sensitive to all.

**Figure 3:**
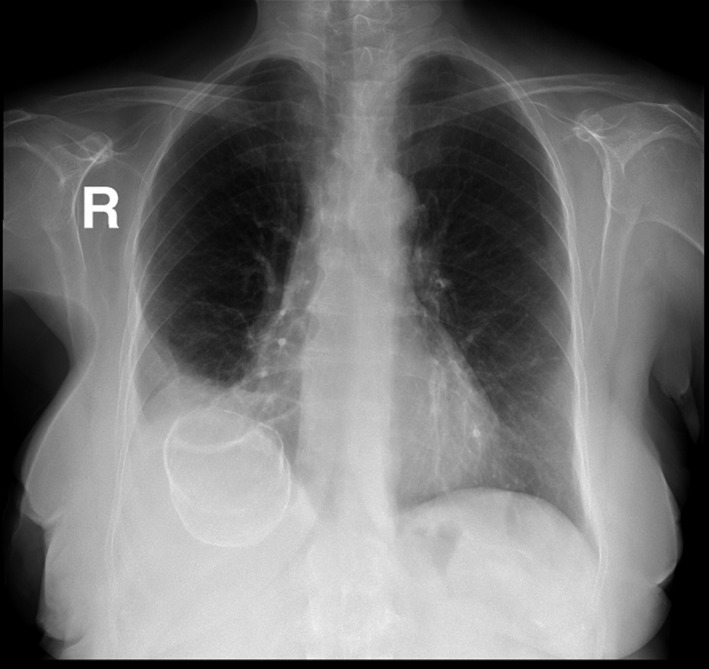
Chest X‐ray showing a calcified lesion in the right upper quadrant of the abdomen.

Chest X‐ray performed (Fig. 3) showed infiltrates associated with loss of volume in the right middle and lower lobes, with elevation of the right hemidiaphragm and a right pleural reaction. A rounded opacity projecting over the right breast and over the lung base was noticed that was described to be a possible calcified lesion in the lung or the liver. At this point, no correlation was performed between the lesion and the presenting clinical picture due to the high suspicion of a wound infection after the partial mastectomy the patient underwent for the fibroadenoma.

Patient underwent a fistulectomy with a right lateral incision of the right breast. Intraoperatively, a stylet was inserted, as an attempt to follow the fistulous tract and remove the surrounding tissue. While following the tract deep into the tissues, the stylet was noticed reaching the intercostal space and going further down beneath the ribs in the extrapleural space. Significant amount of pus was suctioned and drained; a penrose drain was inserted in the wound. No perioperative complications were noted.

**Figure 4:**
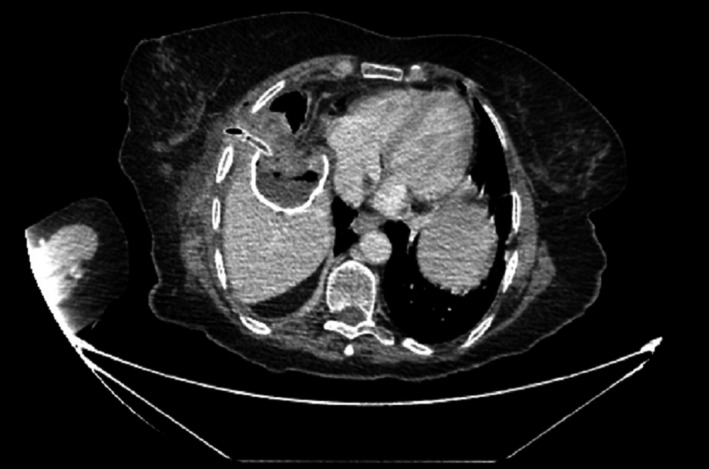
CT scan of the abdomen, showing a calcified hydatid liver cyst, with a fistulous track extending into the thoracic intercostal space.

**Figure 5:**
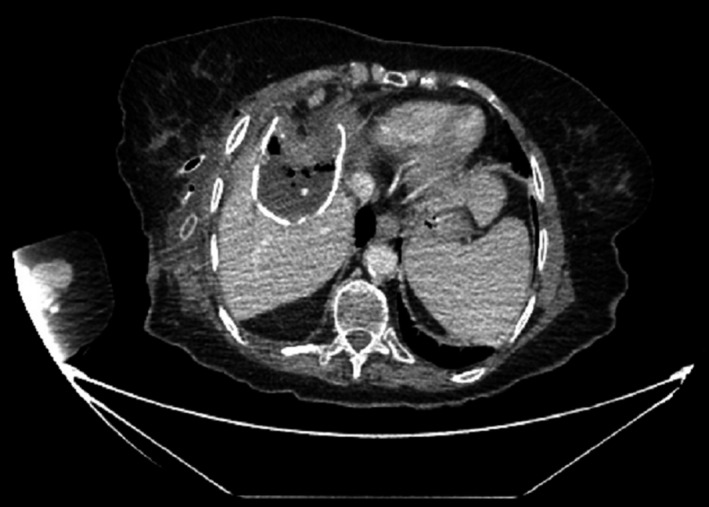
CT scan of the abdomen, showing a calcified hydatid liver cyst, with a fistulous track extending to the thoracic skin level.

At this point, further investigations were necessary for the proper management of this patient. A chest, abdomen, and pelvis CT scan (Figs 4 and 5) were performed because of the unusual finding intraoperatively and showed: 5.5 × 8 × 5.6 cm cyst in the right lobe, involving segments VIII and V of the liver, with wall calcifications. A fistulous tract was also noted extending from the described lesion to the inferolateral margin of the right breast. These findings were compatible with a hydatid cyst with a cutaneous fistula.

The pathology result showed a fistulous tract with no evidence of malignancy from the right breast tissue; inflamed vascular granulation tissue with no evidence of mesothelial lining and no evidence of malignancy.

Conservative management was initiated, with an attempt to drain the cyst, and pull the penrose progressively in order for the tract to heal. Albendazole was started as well. However, after few weeks of daily wound care, no progress was noted regarding the wound's secretion and no signs of imminent fistulous tract closure.

At this point, operative management was decided. Due to the patient's multiple comorbidities, the relatively small left liver and the relatively large cyst, we chose to undergo the least aggressive surgical intervention.

**Figure 6:**
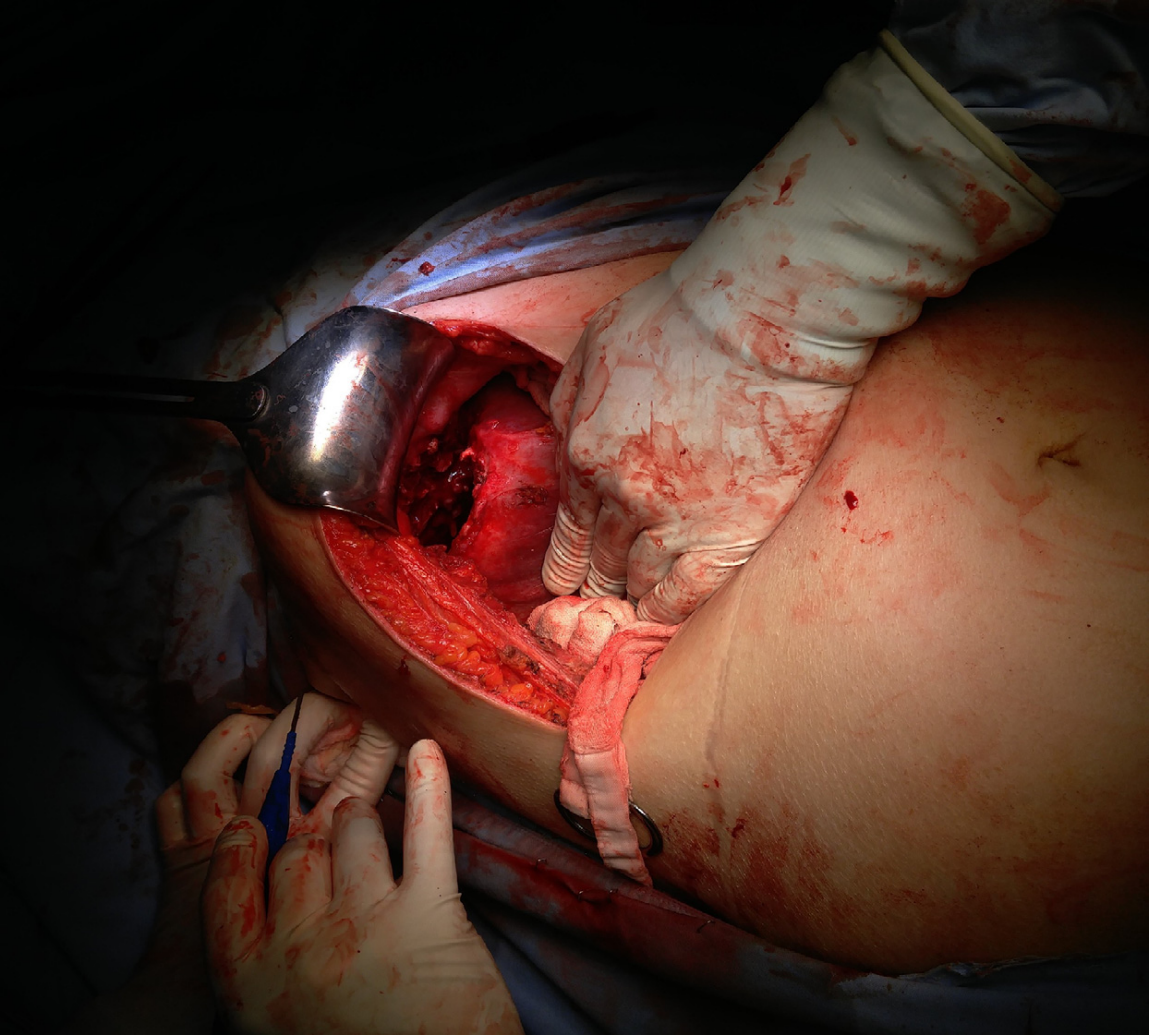
Intraoperative image of the posteriorly located liver hydatid cyst.

**Figure 7:**
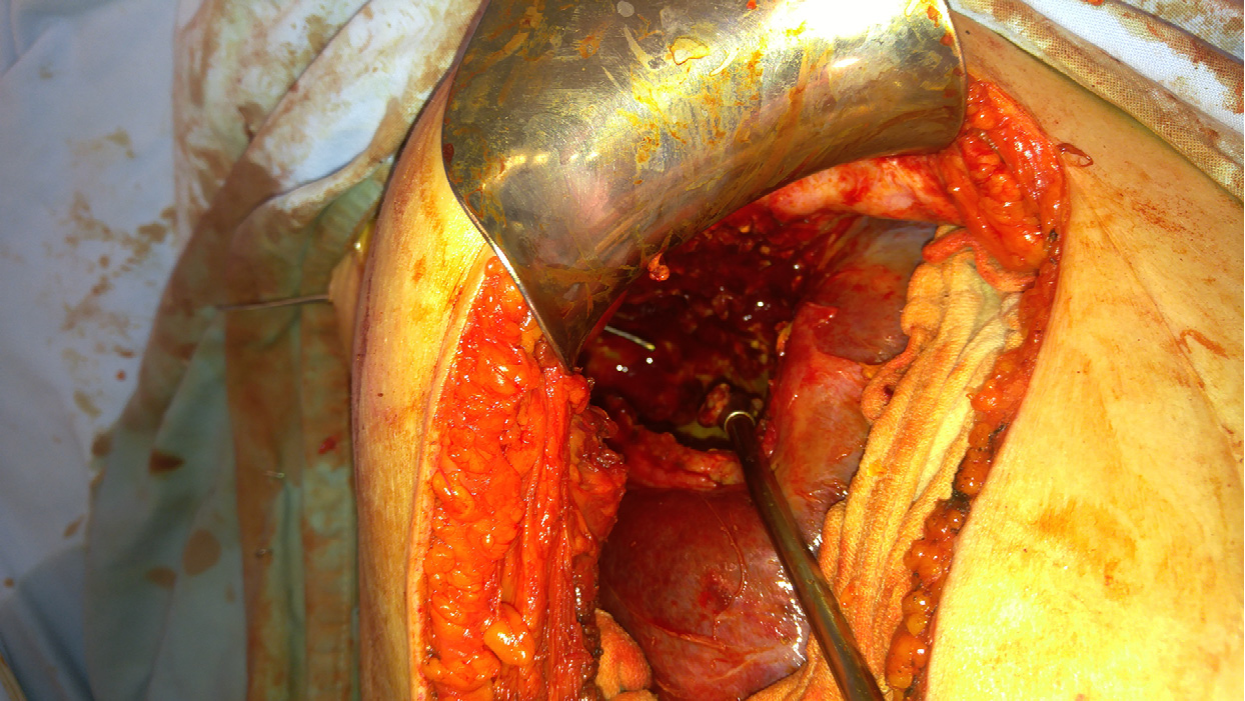
Intraoperative image of the dense cyst content with a stylet inserted in the fistulous tract, one end at the skin level and the other end projecting into the abdomen through the diaphragm.

**Figure 8:**
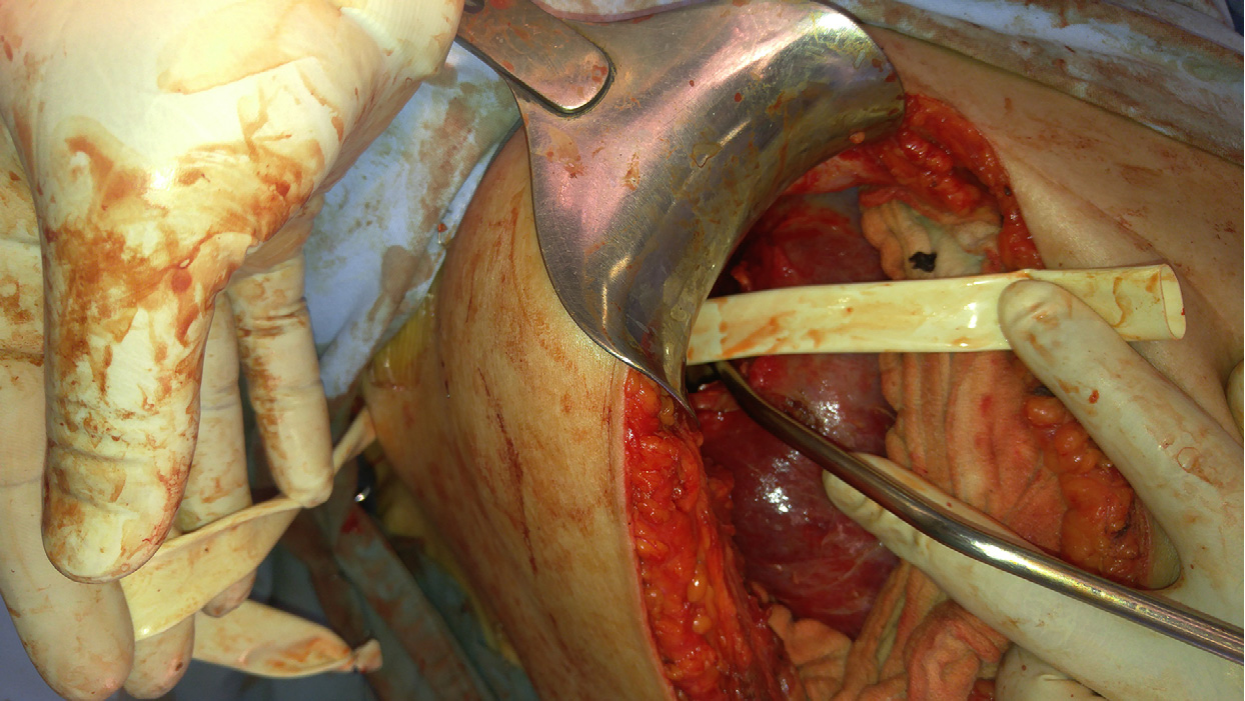
Intraoperative image of the cyst after insertion of the penrose drain in the fistulous tract, one end at the skin level and the other end projecting into the abdomen through the diaphragm.

A right subcostal incision was performed. Liberation and mobilization of the liver was performed until reaching the posteriorly located cyst (Fig. 6). The cyst had a thick calcified wall, adhering circumferentially to the right hemidiaphragm with no intra‐abdominal communication. At this point, the cyst was opened, content was suctioned, and then we proceeded with the liberation of the cyst away from the diaphragm (Fig. 7). The fistulous tract opening in the diaphragm was identified, and a penrose drain was inserted (Fig. 8). The sharp cyst edges were resected circumferentially. The omentum was then liberated preserving its vascular supply, and an omental patch was sewed over the fistulous tract opening in the diaphragm with the penrose drain pulled toward the cutaneous opening of the tract. Two drains were then inserted into the cyst cavity and in the subhepatic space. No perioperative or postoperative complications encountered.

Patient was discharged on postoperative day 5 on Albendazole.

On postoperative day 7, the first intra‐abdominal drain was removed, followed by the penrose drain, which was being progressively pulled out until it was totally out on day 12, and finally the second drain on day 19.

**Figure 9:**
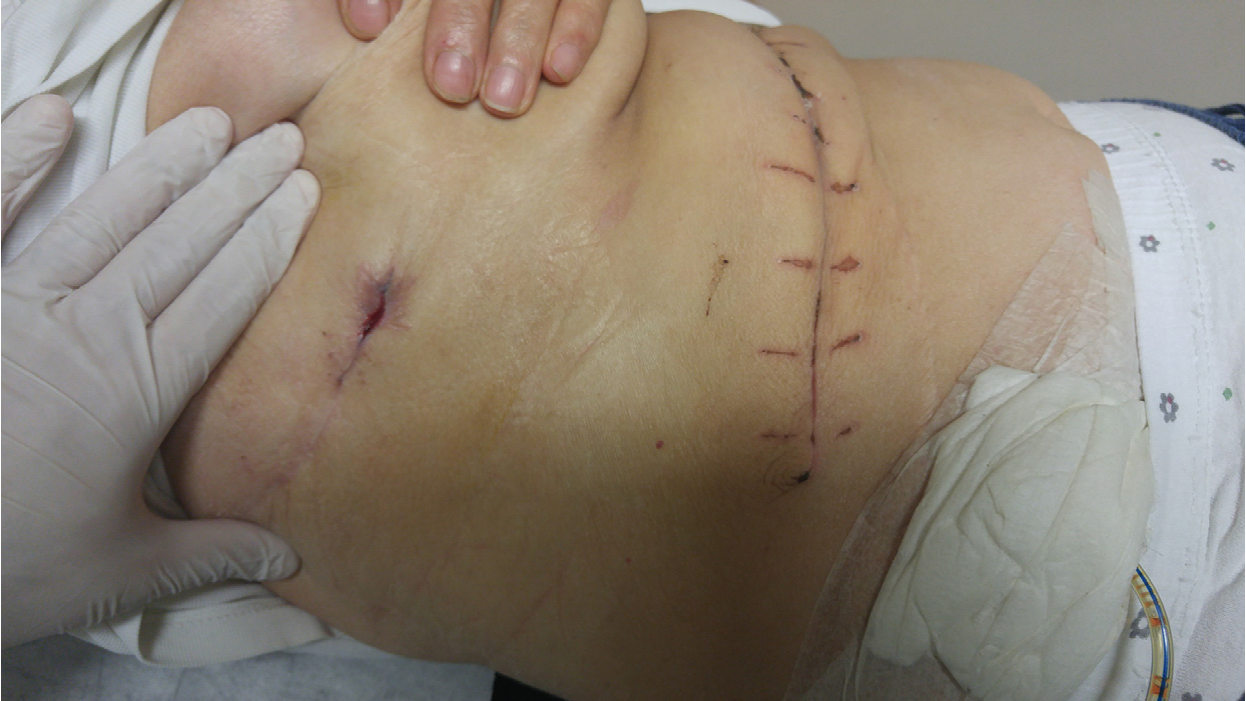
Image showing the patient's healed wounds on follow‐up visit in clinic.

**Figure 10:**
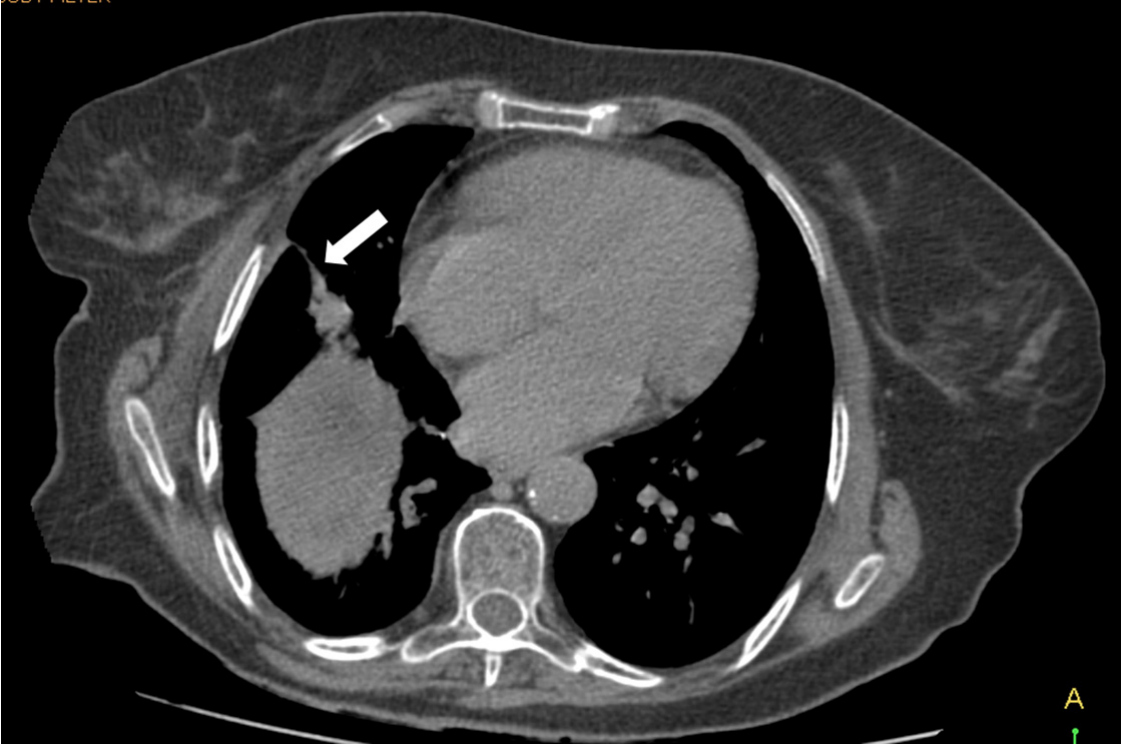
CT scan 2 months postoperatively showing the healed and closed fistulous tract (white arrow).

**Figure 11:**
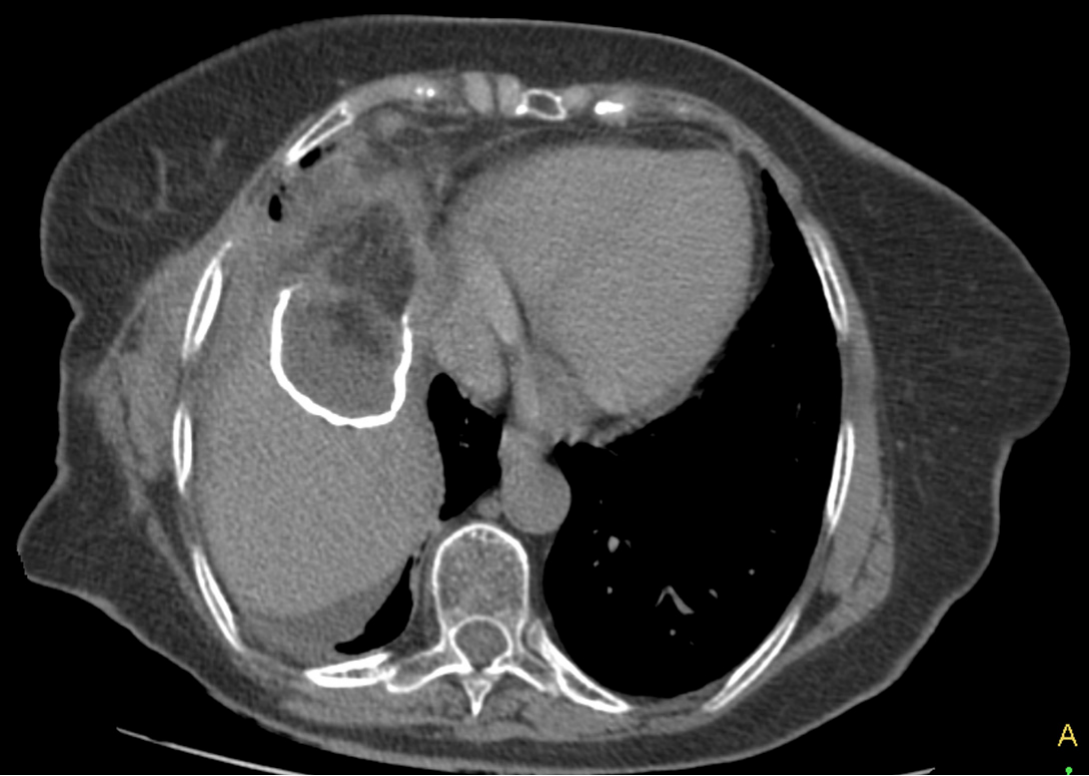
CT scan 2 months postoperatively showing the remnant calcifications of the cyst, the omental patch roofing it, and growing of hepatic tissue around the calcified wall (white arrow).

**Figure 12:**
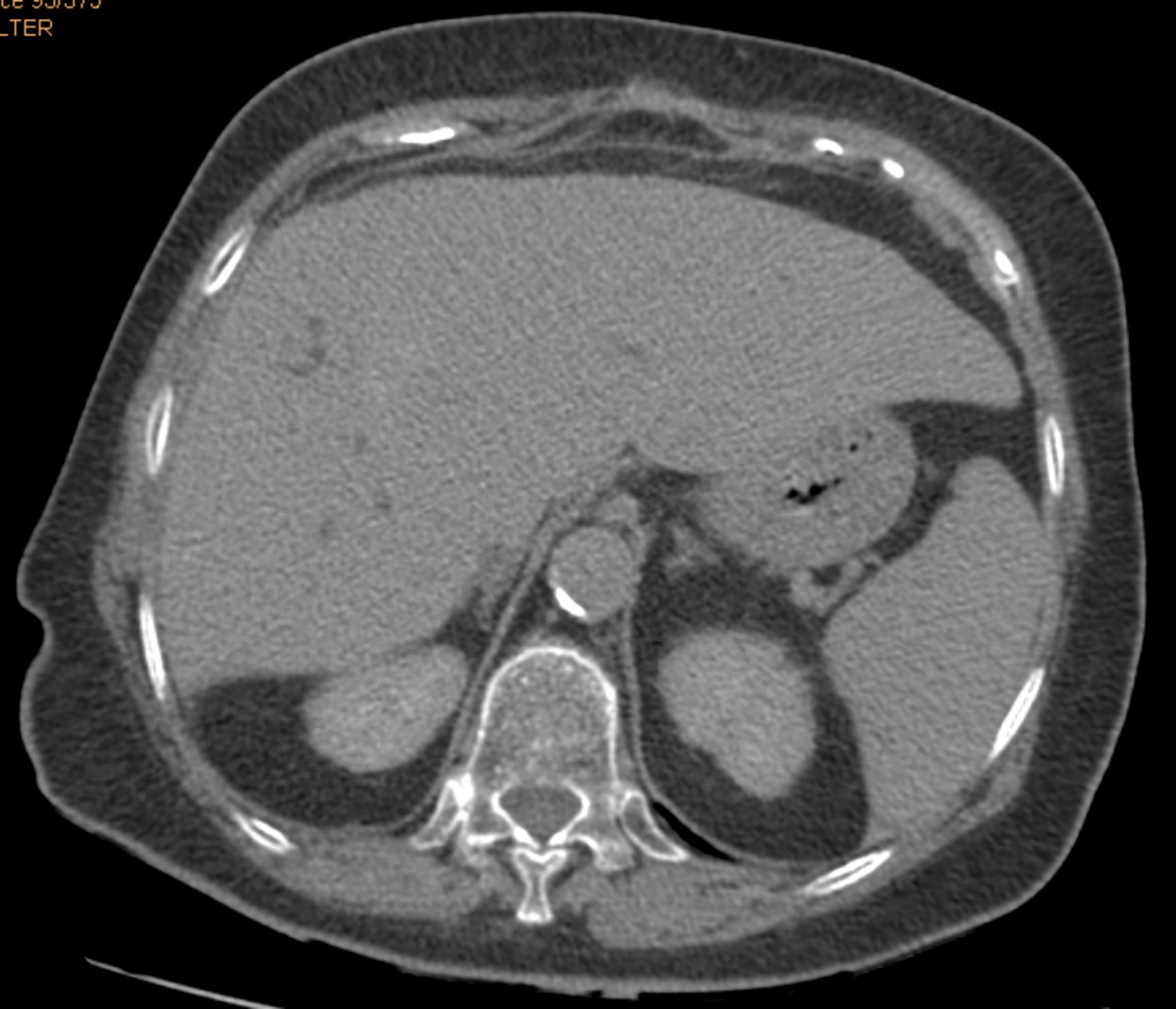
CT scan 2 months postoperatively showing a hypertrophied right liver and no evidence of any intra‐abdominal collections.

Follow‐up visit 1 month postoperatively showed a clinically well‐doing patient with all wounds healed (Fig. 9). At 2 months, a computed tomography scan showed a closed fistulous tract (Fig. 10), some of the calcified cyst wall remnant with normal hepatic tissue growing around it (Fig. 11), no evidence of any intra‐abdominal pathology and a hypertrophied right liver lobe (Fig. 12).

## Discussion

*Echinococcus granulosus*, in the larval form, is the active initiator of hydatid disease in the human species. Canine feces are the main delivery route, although contact with these animals is also a mode of transmission. When eggs from feces‐contaminated vegetables are ingested, they hatch into the small intestines, pass through its wall into the portal vein, where access to liver, lungs or any other organ is guaranteed 1. It occurs in the liver in 52–77% of cases. Uncomplicated cysts tend to be asymptomatic. Symptoms are usually related to a toxic reaction driven by the parasite's presence. Sometimes, local and mechanical effects, or complications, constitute the main presenting symptoms 2. In 10–15% of the time, rupture and communication between the cyst and the biliary tree occur 3. The least‐encountered complication is a hydatid cyst – cutaneous fistula with only 17 cases reported in the literature 4.

The majority of hydatid cysts with cutaneous fistula have one or more of the following features: superficially located cysts, thick, calcified, and showing chronic inflammatory changes. Such characteristics add more sense to the cutaneous communication of these types of cysts 5. Repetitive respiratory movements, associated with friction to a firm and calcified cyst, along with an inflammatory environment, are ideal conditions for nearby tissues erosion and fibrosis, leading progressively to an opening at the skin level.

Furthermore, of the 17 cases reported, none had a cutaneous fistulous tract opening at the level of the breast. Two of the 17 cases were sent on Albendazol only, and succeeded. In two other cases, a conventional treatment was attempted and consisted of cyst drainage through the fistulous tract 6, 7. The remaining cases were operated by fistulous tract excision, followed by resection of the affected segment 4.

In our case, we started with conservative management and failed. When we resolved for surgery, we decided to act the least aggressively by avoiding a thoracotomy, for fistulous tract excision, and a right hepatectomy. The patency of the fistulous tract was maintained by the cyst's secretions. So, on the one hand, we resigned to the idea that breaking the cystofistulous communication might allow the tract to close. This consisted of two steps: division of the cyst's attachment to the diaphragm then preventing the cysto‐fistulous connection with an omental patch. On the other hand, we needed to treat the hepatic hydatid cyst cavity. Scolicidal agent (citramide) was used, cyst content was drained, and partial cystectomy performed. No intrabiliary communication was noted, and a drain was inserted into the cyst.

Omentoplasty, cyst drainage, and fistulous tract drainage revealed successful in the management of hydatid cyst with spontaneous cutaneous fistula. Such an intervention can stand as an alternative when conservative treatment fails, and a minimally aggressive procedure is required.

## Authorship

AEA: did the literature review and corrected the article. EC: wrote the article and organized the figures, tables, and legends. MY: did the data collection and obtained the patient's consent. IH and MEK: performed the operation and reviewed the written article. WD and AEA: assisted in the surgical intervention.

## Conflict of Interest

The authors declare no potential conflict of interest.
